# Novel High-Viscosity Polyacrylamidated Chitosan for Neural Tissue Engineering: Fabrication of Anisotropic Neurodurable Scaffold via Molecular Disposition of Persulfate-Mediated Polymer Slicing and Complexation

**DOI:** 10.3390/ijms131113966

**Published:** 2012-10-29

**Authors:** Pradeep Kumar, Yahya E. Choonara, Lisa C. du Toit, Girish Modi, Dinesh Naidoo, Viness Pillay

**Affiliations:** 1 Department of Pharmacy and Pharmacology, Faculty of Health Sciences, University of the Witwatersrand, 7 York Road, Parktown, 2193, Johannesburg, South Africa; E-Mails: pradeep.kumar@wits.ac.za (P.K.); yahya.choonara@wits.ac.za (Y.E.C.); lisa.dutoit@wits.ac.za (L.C.T.); 2 Department of Neurology, Division of Neurosciences, Faculty of Health Sciences, University of the Witwatersrand, 7 York Road, Parktown, 2193, Johannesburg, South Africa; E-Mail: gmodicns@mweb.co.za; 3 Department of Neurosurgery, Division of Neurosciences, Faculty of Health Sciences, University of the Witwatersrand, 7 York Road, Parktown, 2193, Johannesburg, South Africa; E-Mail: dinesh.naidoo@wits.ac.za

**Keywords:** neural tissue engineering, polymer composite, polyacrylamidated chitosan, potassium persulphate, polymer grafting, neurodurable scaffold, molecular modeling and simulation

## Abstract

Macroporous polyacrylamide-grafted-chitosan scaffolds for neural tissue engineering were fabricated with varied synthetic and viscosity profiles. A novel approach and mechanism was utilized for polyacrylamide grafting onto chitosan using potassium persulfate (KPS) mediated degradation of both polymers under a thermally controlled environment. Commercially available high molecular mass polyacrylamide was used instead of the acrylamide monomer for graft copolymerization. This grafting strategy yielded an enhanced grafting efficiency (GE = 92%), grafting ratio (GR = 263%), intrinsic viscosity (IV = 5.231 dL/g) and viscometric average molecular mass (MW = 1.63 × 10^6^ Da) compared with known acrylamide that has a GE = 83%, GR = 178%, IV = 3.901 dL/g and MW = 1.22 × 10^6^ Da. Image processing analysis of SEM images of the newly grafted neurodurable scaffold was undertaken based on the polymer-pore threshold. Attenuated Total Reflectance-FTIR spectral analyses in conjugation with DSC were used for the characterization and comparison of the newly grafted copolymers. Static Lattice Atomistic Simulations were employed to investigate and elucidate the copolymeric assembly and reaction mechanism by exploring the spatial disposition of chitosan and polyacrylamide with respect to the reactional profile of potassium persulfate. Interestingly, potassium persulfate, a peroxide, was found to play a dual role initially degrading the polymers—“polymer slicing”—thereby initiating the formation of free radicals and subsequently leading to synthesis of the high molecular mass polyacrylamide-grafted-chitosan (PAAm-g-CHT)—“polymer complexation”. Furthermore, the applicability of the uniquely grafted scaffold for neural tissue engineering was evaluated via PC12 neuronal cell seeding. The novel PAAm-g-CHT exhibited superior neurocompatibility in terms of cell infiltration owing to the anisotropic porous architecture, high molecular mass mediated robustness, superior hydrophilicity as well as surface charge due to the acrylic chains. Additionally, these results suggested that the porous PAAm-g-CHT scaffold may act as a potential neural cell carrier.

## 1. Introduction

Chitosan, a biocompatible and biodegradable natural polysaccharide, has been extensively studied for its potential as a biomaterial for tissue engineering and drug delivery applications [1,2]. In addition to the pristine chitosan, graft-copolymerized chitosan and modified chitosan derivatives have been extensively researched and reviewed for various biomedical applications [3–7]. Recently, this biopolymer has shown promise in neural tissue engineering in the form of nerve guidance conduits (NGCs) and scaffold with specific improvements in nerve cells’ attachment, differentiation, and growth after derivatization or blending with other polymers such as poly-l-lysine [8]. Additionally, poly(acrylamide) individually as well as in combination with other polymers such as poly(urethane) has been proposed to be valuable in neural tissue engineering as a cell carrier with sustained bioactive-release properties [2,9,10].

Graft copolymers of chitosan/derivatized chitosan and acrylamide monomer have been extensively studied in terms of their synthesis and evaluation in controlled drug release [11,12], adsorption separation of azo dyes [13], metal ions [14], or proteins [15] and as flocculants [16]. Conventionally acrylamide monomers are grafted onto chitosan using redox systems, ceric-ions or microwave-assisted reactions. Among these, redox systems, and particularly potassium or ammonium persulphate, are the most commonly used initiator systems for grafting of acrylamide monomer (AAm) onto chitosan [3]. The persulfate-chitosan redox system radically initiates the polymerization of AAm leading to formation of a graft copolymer: polyacrylamide-graft-chitosan (AAm-g-CHT) and a homopolymer: polyacrylamide [14,17]. However, this homopolymer formation remains the main constraint in commercializing the procedures due to the low molecular mass of the graft copolymers and low grafting yield [9].

The graft co-polymerization of chitosan with “presynthesized” polymers such as polyethylene glycol (PEG) and polyethylenimine (PEI) forming PEG-g-chitosan [18] and PEI-g-chitosan [19] have already been reported where intact functionalized polymers instead of monomers were used for modification of chitosan. In comparison to conventional grafting techniques using monomers, the advantages of using end-group modified presynthesized polymers is the absence of residual monomers and improved side-chain molecular mass control.

However, a very high molecular mass polymer may inhibit the grafting reaction due to steric hindrances and reduced molecular mobility as a result of a higher viscosity. To our knowledge, the synthesis of polyacrylamide-(the native polymer)-g-chitosan via a graft copolymerization reaction has not been reported although polyacrylamide has been used with chitosan as a blend forming an interpenetrating polymer network [20]. It is well acknowledged and established that polyacrylamide undergoes degradation in the presence of persulphate ions leading to the formation of high molecular mass polyacrylamide free radicals that may act as monomer radicals [21,22]. This is also true for chitosan, as persulphates result in the cleavage of polymer chains [23–25]. Hence it is predicted that the presence of persulphates may affect the grafting of polyacrylamide directly onto chitosan chains and thereby completing the graft copolymerization reaction. In accordance with the discussion above, the possibility of producing homopolymers/monomers will be extremely low and the steric hinderences and molecular immobility will be reduced due to polymer slicing by persulphate ions.

Therefore the aim of this work is to prove for the first time the novel strategy of utilizing PAAm for grafting onto CHT and show the applicability of polyacrylamidated chitosan in neural engineering. In addition, this study also display the uniqueness of synthesizing polyacrylamide-g-chitosan efficiently without the use of monomers, as well as elucidating the reactional profile and structure of the free-radical grafting reaction on the basis of Static Lattice Atomistic Simulations. The reaction mechanism of the grafting process was investigated in order to determine the appropriate synthesis conditions. For convenience, graft copolymer (polyacrylamide-g-chitosan) synthesized via acrylamide (monomer) and polyacrylamide (polymer) will be denoted as AAm-g-CHT and PAAm-g-CHT, respectively, throughout this study. In coherence with the above, comparative scaffold will be fabricated using AAm-g-CHT and PAAm-g-CHT and the effect of the graft-copolymerization on the viscosity/molecular mass and porosity will be tested utilizing PC12 neuronal cells to asserting the applicability of the newly fabricated grafted biopolymer.

## 2. Results and Discussion

### 2.1. Copolymer Synthesis, Grafting Parameters and Molecular Mass Analysis

Graft copolymerization of CHT with AAm and PAAm was achieved in the presence of potassium persulfate catalyzed free-radical polymerization. For the synthesis of PAAm-g-CHT, the mass ratio of CHT:PAAm (1:1) was four times lower than that of CHT-AAm (1:4) in CHT-g-AAm due to the increased viscosity of the CHT/PAAm solution as a result of the high molecular mass of PAAm. The quantity of KPS used in the CHT-g-PAAm reaction was much higher because as it was involved in the degradation of PAAm prior to grafting. The viscosity of both solutions decreased drastically in the presence of KPS when added to the reaction mixtures [11]. The presence of KPS affected the degradation of the polymers from free radicals produced by CHT and PAAm. The high molecular mass free radicals of PAAm subsequently acted as a functionalized polymer chain (as in the case of “presynthesized” polymers such as PEG and PEI forming PEG-g-CHT [18] and PEI-g-CHT [19], respectively) with a free radical at one end available to covalently react with the CHT free radical. These polymeric radicals combine to form the limiting homopolymer PAAm as previously reported in the case of AAm-g-CHT. The complete mechanism of this reaction is summarized and molecularly deduced by SLAS later in this paper.

The comparative profiles of the grafting parameters such as the intrinsic viscosities and molecular mass of the graft copolymers are listed in Table 1. Although, the volume of PAAm used in the synthesis of CHT-g-PAAm was much less than AAm used in synthesizing AAm-g-CHT, it was evident from the data that the grafting efficiency and grafting ratio were higher in the case of PAAm-g-CHT. Additionally, PAAm-g-CHT had a higher molecular mass than AAm-g-CHT. This unique finding may be due to two possible reasons: Firstly, the degradation of PAAm resulted in long-chain functional polymers that directly attached to CHT instead of inherent chain propagation onto CHT, and secondly, the low or non-formation of a homopolymer (PAAm).

### 2.2. Investigation of Polymeric Structural Transitions

**Figure 1:**
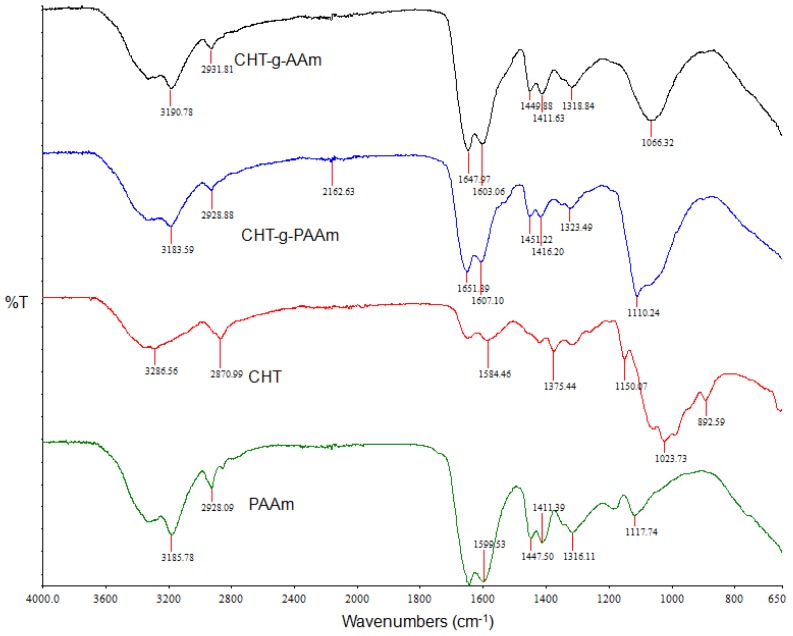
Attenuated Total Reflectance-FTIR (ATR-FTIR) spectra of Polyacrylamide (PAAm), Chitosan (CHT), CHT-g-PAAm and CHT-g-AAm (ascending order).

ATR-FTIR spectroscopy was employed to confirm the graft copolymerization using PAAm and compare the IR spectra of CHT with that of the grafted products and PAAm. It is evident from Figure 1 that CHT displayed strong bands at 1023 cm^−1^, 1081 cm^−1^, and 1375 cm^−1^, due to O–H bending, C–O stretching, and C–N stretching, respectively, which are characteristic of a polysaccharide molecule. Additionally the band in the range of 3200–3450 cm^−1^ (at 3286 cm^−1^ and 3414 cm ^−1^) depicted the O–H stretching vibration, N–H extension vibration, and intermolecular H-bonds. A characteristic peak of C=O integration of 1650 cm^−1^ due to partial deacetylation and characteristic peaks of amino groups at 3400 cm^−1^ and 1320 cm^−1^ were also observed. For PAAm, the peaks around 1665 cm^−1^ and 1599 cm^−1^ can be assigned to the amide I and II bands, respectively. The FTIR spectrum of the synthesized AAm-g-CHT showed typical bands at 1647 cm^−1^ (amide I) and 1603 cm^−1^ (amide II) due to grafted PAAm chains onto CHT.

Additionally, the IR spectra of PAAm-g-CHT showed a peak at 1449cm^−1^, due to the C–N stretching, which supported the occurrence of the grafting reaction. Furthermore, the appearance and intensification of the two peaks at 1411 cm^−1^ and 1318 cm^−1^ for the primary NH_2_ groups confirmed that PAAm chains grew on the CHT backbone. The stretching peak at 3414 cm^−1^ shifted to a longer wavenumber (approximately 3428 cm^−1^) and was of a reduced intensity with broadness (overlapping of O–H stretching of CHT and N–H stretching of amide groups at PAAm grafts). Reduced intensity of this peak with respect to CHT proved that an appreciable quantity of O–H and N–H groups in CHT had been grafted with the PAAm chains. These results are coherent with earlier reports on graft copolymerization of PAAm onto CHT [13,14,16,26–28]. For the PAAm-g-CHT, FTIR spectra showed major peaks at 3183 cm^−1^, 1651 cm^−1^, 1607 cm^−1^, 1451 cm^−1^, 1416 cm^−1^ and 1323 cm^−1^ corresponding to 3190 cm^−1^, 1647 cm^−1^, 1603 cm^−1^, 1449 cm^−1^, 1411 cm^−1^ and 1318 cm^−1^ peaks of AAm-g-CHT, respectively. With the incorporation of PAAm instead of AAm in the grafting strategy, the intensity of all peaks increased significantly from the AAm-g-CHT to PAAm-g-CHT. This result suggested that more amide (I) groups were introduced onto the CHT backbone; that is the polymer chain of grafted PAAm was longer in PAAm-g-CHT forming a high molecular mass graft copolymer. The occurrence of a peak at 1110 cm^−1^ and a shoulder at 889 cm^−1^ further proved that PAAm-g-CHT had fewer but longer PAAm chains (exposing more PAAm and the CHT backbone) compared with AAm-g-CHT that had a large number of short PAAm chains. Additionally, the increase in ratio of the peak intensities at 1651 cm^−1^ to 1607 cm^−1^ may be due to an increase in the grafting efficiency. The increase in the number of amide groups, as revealed by FTIR studies, may render more hydrophilicity to the PAAm-g-CHT as well as an increase in surface charge leading to enhanced neurocompatibility and adhesion—the prerequisites for a neural scaffold [10]. Furthermore, the longer PAAm chains will render high “crosslinkability-induced” robustness providing an optimal platform for the mechano-sensitive PC12 neuronal cells as mentioned earlier in this paper.

### 2.3. Comparison of Thermally-Induced Transitions of the Grafted Copolymers

**Figure 2:**
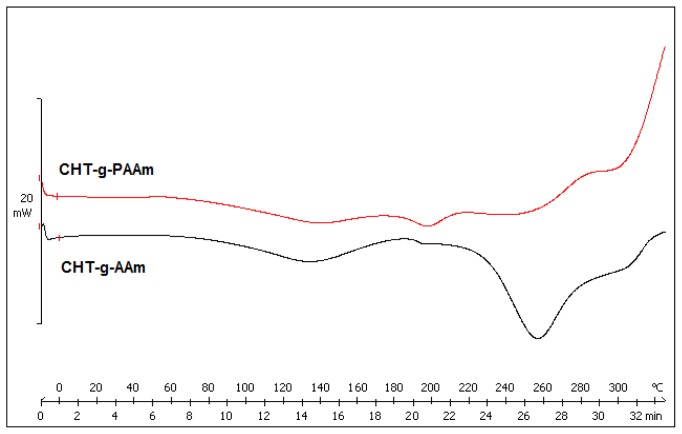
DSC thermogram of graft copolymers in the second heating run.

DSC was used to measure and compare the thermal transitions inherent to the synthesized copolymers as a function of temperature thereby directly measuring different heat flows between the reference (AAm-g-CHT) and sample (PAAm-g-CHT) [29]. The DSC curves of the copolymers are depicted in Figure 2 revealing three thermal transitions. Firstly, for CHT-g-AAm, the thermal transition at 132 °C represents the structural relaxation associated with the glass transition temperature (*T*_g_) which corresponds to the *T*_g_ of PAAm (127.3 °C). The higher *T*_g_ may be due to the covalent coupling of PAAm to the CHT backbone via graft copolymerization [13]. The second thermal transition at 197 °C corresponds to the *T*_g_ of CHT (203 °C) as reported by Sakurai and co-workers (2000) [30]. There is considerable debate over the exact value of the structural relaxation associated with the *T*_g_ of chitosan as discussed by Duarte, Mano and Reis (2010) [31]. The lowering of the *T*_g_ of chitosan could be due to the inclusion of PAAm on the CHT backbone in coherence with Fox’s theory [30]. The DSC thermogram of CHT-g-AAm also exhibited an endothermic peak at 256 °C that was attributed to the melting point (*T*_m_) of the grafted polymer moiety (PAAm chains) [32].

In the case of PAAm-g-CHT, the above-mentioned thermal transitions appeared at 136 °C, 197 °C and 254 °C corresponding to 132 °C, 197 °C and 256 °C of AAm-g-CHT, respectively. Although the thermal peaks differed in terms of their onset, endset and depth, the thermal properties of the polymer components were not significantly different in terms of eventual peak values which confirmed the comparability of the structural profile of the synthesized graft polymers as explained in the ATR-FTIR discussion. The decrease in *T*_g_ of PAAm in PAAm-g-CHT may be due to an increase in chain length [33]. The increase in chain length may further affect the cellular response based on robustness as explained in earlier sections. Furthermore, the decrease in peak intensity in the case of PAAm-g-CHT displayed a less crystalline/more amorphous character of the newly synthesized PAAm-g-CHT copolymer due to more exposure of the CHT chains. From neural tissue engineering prospective, the amorphous nature of the PAAm-g-CHT scaffold may further render superior neurocompatibility and invasiveness as the cell adhesion, growth and proliferation are inversely proportional to the crystallinity of the scaffold architecture [34].

### 2.4. Morphological Characterization and Quantitative Image Processing Analysis

**Figure 3:**
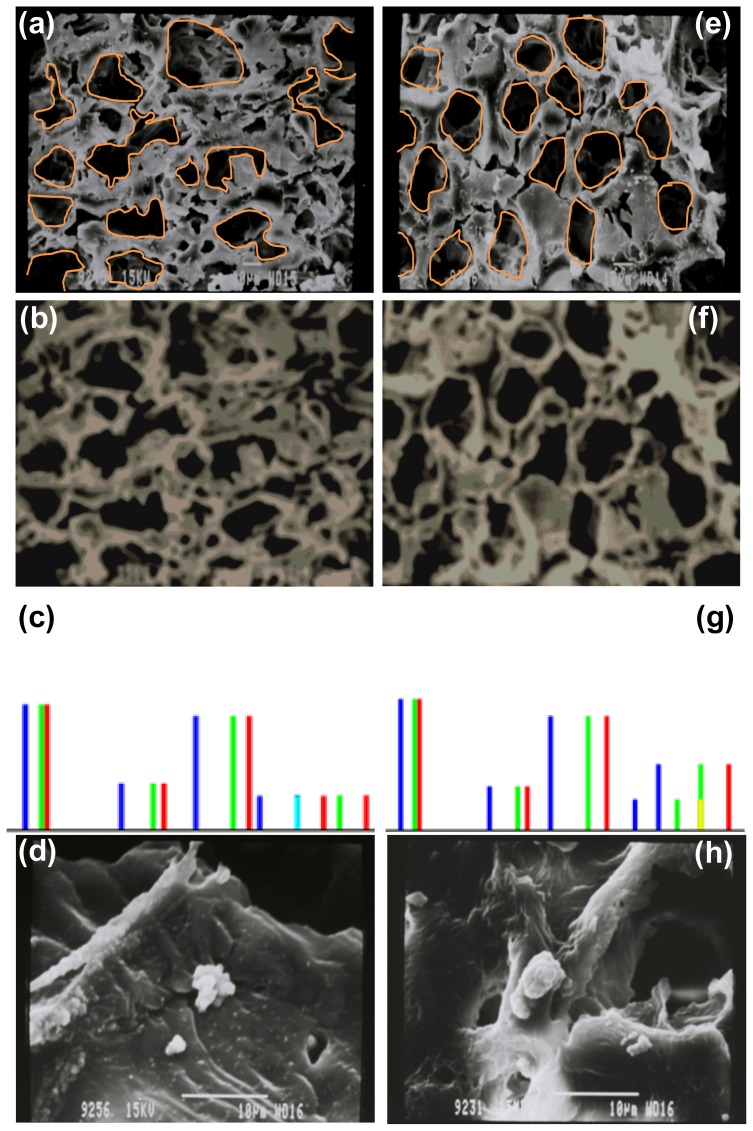
Scanning electron micrographs, ColorQuantized images, ImageHistograms and Cytocompatibility of CHT-g-AAm (**a**, **b**, **c**, **d**); and CHT-g-PAAm (**e**, **f**, **g**, **h**), respectively.

Image Processing was performed on 16-bit greyscale photomicrograph images obtained by SEM of the AAm-g-CHT and PAAm-g-CHT scaffold (Figure 3). Thresholding of crude images restricted data capture and therefore intermediary image-processing steps were employed to equilibrate the background and contrast fields between the polymeric architecture, pore structure and the non-Focus areas of the image. Images in TIFF format were computatively imported using Equation 1 and converted to Mathematica™ 8.0 format.

(1) SEMimage=Import[“X:\\directory\\folder\\filename.tif”]

The first processing step, blurring the image, provided a blurred version of the image obtained by convolving the image with a low pass filter. This was important for the quantitative mapping of the images. In this work, the extent of fuzziness was increased by increasing the pixel radius (r) to 10 quantitative points without compromising the image details. This was achieved in Mathematica™ 8.0 by applying a custom blurring function (Equation 2).

(2) BLURimage=Blur[SEMimage,10]

After blurring the image, the next step was to ColorQuantize the blurred image at a value of 5 that provided an approximation to an image that utilized 5 distinct colors. This was achieved by applying a custom ColorQuantize function (Equation 3) (Figure 3 ColorQuantized).

(3) CQimage=ColorQuantize[BLURimage,5]

The third step involved analyzing the ColorQuantized images by histogram plots of the pixel levels for each channel in the image (Figure 3). Furthermore, separate histograms for each color channel were constructed, by applying an ImageHistogram function for default and separated algorithms using Equation 4.

(4) Histogram=ImageHistogram[CQimage,Appearance→“Transparent”]

Analysis of the histogram plots are essential for optimizing the threshold and discriminating between different morphological features observed on the SEM of the AAm-g-CHT and PAAm-g-CHT scaffold. An assumption was that the probability of finding intensity at a pore-fiber boundary would be equal for both features. Figure 3c, CHT-g-AAm, displayed a tetramodal distribution having peaks at four different voxel intensities representing voxels for air-filled deep pores and pores reaching the second or third layer of the polymeric structure (smaller peaks), as well as solid polymeric architecture at different layers in order of increasing linear absorption coefficients (larger drifts). For CHT-g-PAAm (Figure 3e), the histogram plot of the SEM image represented a pentamodel distribution having all four voxel peaks of CHT-g-AAm and one added peak of near average intensity. The new threshold generation implied that the intensity was more in congregation with the porous structure than that of the polymeric solid structure. These transitions toward air-filled pores may be attributed to an increase in graft co-polymer chain lengths (decreased solid area) leading to formation of larger uniform pores resulting in an increase in the porosity.

The SEM images shown in Figure 3d,h, 2 days after cell-seeding, clearly depict the effect of the grafting strategy on the cellular response. During SEM analysis of AAm-g-CHT, no PC12 neuronal cells were present within the scaffold with a small population existing only on the surface of the scaffold as shown in Figure 3d. Interestingly, the PAAm-g-CHT displayed the presence of PC12 cells inside the scaffold and were much more developed (Figure 3h). However, no significant axonal extension was observed in both cases over the 2 days. According to Chung and Park (2007), “an ideal polymeric scaffold requires several structural and chemical features: (i) a three-dimensional architecture with a desired volume, shape, and mechanical strength, (ii) a highly porous and well interconnected open pore structure to allow high cell seeding density and tissue in-growth, (iii) chemical compositions such that its surface and degradation products are biocompatible causing minimal immune or inflammatory responses, and (iv) their degradation rate finely tuned in a pattern such that it provides sufficient support until the full re-growth of impaired tissues” [35]. The present scaffold structure fabricated from PAAm-g-CHT successfully delivers on the first three aspects reported by Chung and Park (2007) with the fourth requiring further research. The applicability of PAAm-g-CHT to neural tissue engineering can be attributed to its anisotropic porous scaffold architecture leading to cell and tissue infiltration into the biopolymeric scaffold. Furthermore, due to the high molecular mass of PAAm-g-CHT as compared to AAm-g-CHT, it may result in multiple crosslinkages per polymer chain, essentially producing a more robust network with increased tensile moduli and stiffness (at lower polymer concentrations).

### 2.5. Static Lattice Atomistic Simulations

**Figure 4:**
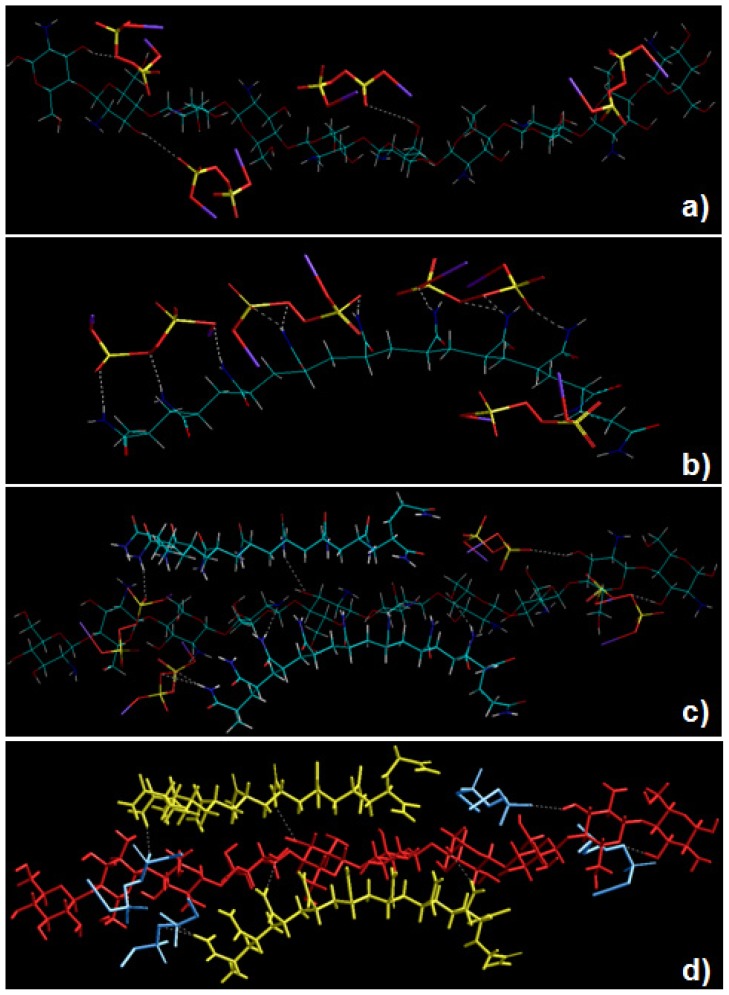
Energy minimized geometrical preferences of the molecular complexes derived from molecular mechanics calculations: (**a**) Chitosan (sticks)-KPS (tube); (**b**) Polyacrylamide (sticks)-KPS (tube); (**c**) Chitosan-PAAm-KPS and (**d**) Chitosan(red)-PAAm(yellow)-KPS(blue). Color codes for elements are: Carbon (cyan), Nitrogen (blue), Oxygen (red), Potassium (purple) and Hydrogen (white).

**Scheme 1:**
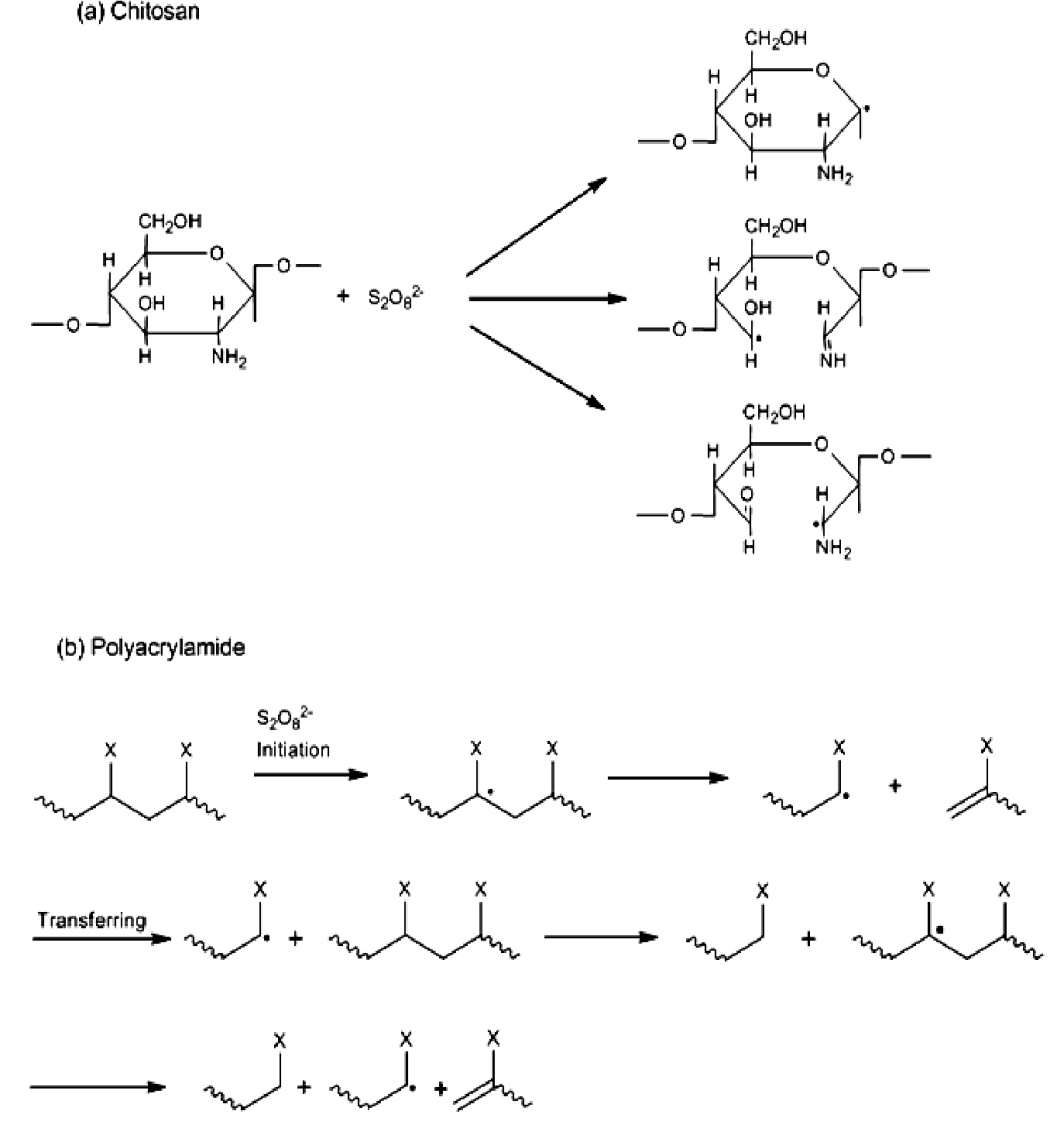
Schematic representation of chain degradation—“polymer slicing”—and free radical formation of (**a**) Chitosan; and (**b**) Polyacrylamide (X represents the group of –CONH_2_) in the presence of persulphate ions.

**Scheme 2:**
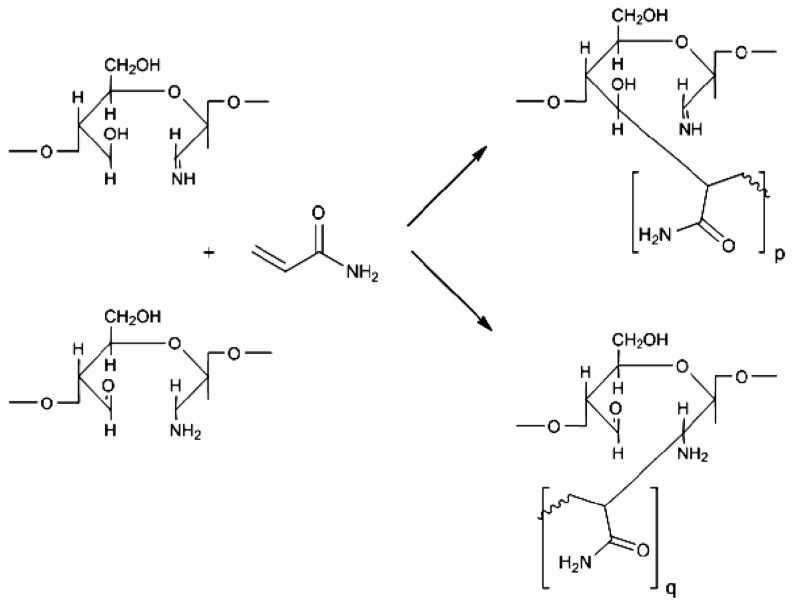
Schematic representation of radical induced graft copolymerization—“polymer complexation”—of AAm to chitosan in the presence of persulphate ions.

**Scheme 3:**
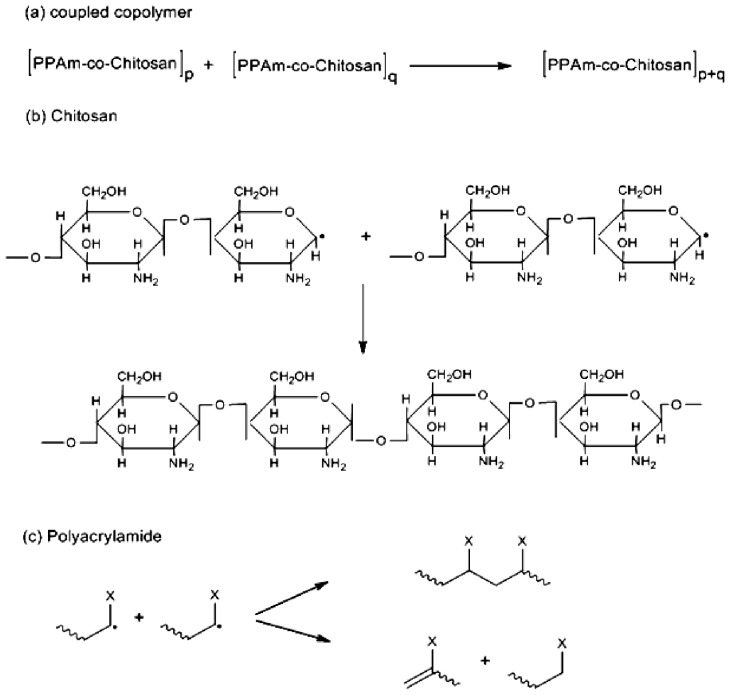
Schematic representation of termination of the graft copolymerization leading to formation of (**a**) coupled graft copolymer; (**b**) chitosan polysaccharide; and (**c**) polyacrylamide homopolymer (X represents the group of –CONH_2_), in the presence of persulphate ions.

**Scheme 4:**
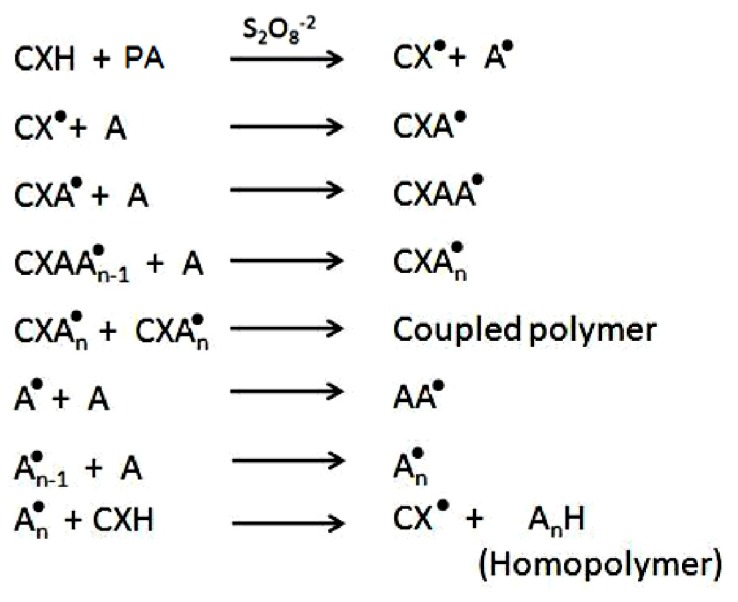
Schematic representation of mechanism summary of CHT-g-PAAm in the presence of persulphate ions, where, C = Chitosan; X = O or N for (PAAm-co-Chitosan)_p_ or (PAAm-co-Chitosan)_q_, respectively; PA = Polyacrylamide; A= Acrylamide.

Molecular Mechanics (MM) describes the energy of a molecule in terms of a simple function which accounts for distortion from ideal bond distances and angles, as well as for non-bonded Van der Waals and Coulombic interactions. To corroborate the experimental results with added confidence, global energy minimizations were employed, to demonstrate the non-bonding electro- and structure-selective binding of the polymeric moieties, CHT and PAAm in the presence of KPS. In this work, we established a novel grafting strategy to synthesize PAAm-g-CHT (Schemes 1–4). Table 2 and Figure 4 display the results of computations undertaken in vacuum. The molecular tectonics of coupled-complexed-copolymer in this study was found to be affected by various types of non-bonding attractive interactions such as Van der Waals contacts, H-bonds and electrostatic interactions. It is evident from Figure 4a, that KPS interacted with C-3 and C-6 of the CHT glucosamine polysaccharide moiety resulting in “polymer slicing” of C2–C3 and the β-(1–4)-linkage, respectively, which further lead to formation of free radical sites as displayed in Scheme 1a. Similarly, KPS interacted with the –CONH_2_ group of PAAm (Figure 4b) and initiated the degradation of the polymer chain— “polymer slicing”—into smaller fragments via the formation of free radicals as displayed in Scheme 1b. In addition, the steric energy profiles of CHT-KPS_4_ (Δ*E* = −35.235 kcal/mol) and PAAm-KPS_4_ (Δ*E* = −51.762 kcal/mol) confirmed the inherent binding stability of the complexes through non-bonding interactions namely electrostatic (Δ*E* = −32.394 kcal/mol) and H-bonding (Δ*E* = −3.039 kcal/mol), respectively. Both the complexes were also equally stabilized by London dispersion forces. It is evident from the reactional profiles that CHT and PAAm provide an abundance of reactive functional groups such as –COO^−^, –NH^3+^, –OH, –CONH_2_, and –NH_2_[36]. The presence of such ionic functional groups may further provide a conductive surface environment for the growth and proliferation of the neural architecture on account of their mixed hydrophillicity/hydrophobicity and varied surface-to-charge ratio.

To further elucidate the grafting mechanism of PAAm and CHT, computations were executed by simulating CHT, PAAm and KPS in a closed system as shown in Figure 4c and 4d. Apparently, PAAm molecules formed bonds with the free radical sites generated in the CHT ring *i.e.*, coupling occurred through –CONH_2_ carrying carbon of PAAm and the C2 (–NH_2_) and C3 (–OH) of CHT—“polymer complexation”—as evident from Figure 4c. The mechanistic structural profiles pertaining to this coupling is explained in Scheme 2. Noticeably, these are the same sites through which KPS interacted with both polymers. The total energy profile of CHT-PAAm_2_-KPS_4_, also favored the formation of the grafted copolymer with impressive highly stabilized values of Δ*E* = −92.199 kcal/mol, Δvdw = −86.158 kcal/mol, *E*_H-bonding_ = −5.927 kcal/mol and *E*_electrostatic_ = −33.481 kcal/mol. This energy stabilization owing to the presence of the bulky carboxylic and amide groups in sterically favorable equatorial positions with H-atoms occupying axial positions strengthened the proposal of employing polyacrylamidated chitosan for neural engineering where a favorable interaction of the biological system with the biomaterial depends on the copolymer’s hydrophobicity, feature size, radius of curvature, charge and coatings. These phenomena are out of the scope of this study and require further investigation.

Upon the termination of the grafted copolymer formation, the free radicals of the respective polymers combine as shown in Scheme 3. This further supports our approach of using a pre-synthesized polymer (PAAm) and the possibility of having monomers in the final product is minimized. Figure 4d depicts that the energy minimized Van der Waals radii structure of CHT-PAAm_2_-KPS_4_ was due to the flexibility of the PAAm chain, the relevant segments of polymeric rearrangement to form a remarkable structure fit between the –NH_3_^+^, –COO^−^ and –OH^−^ ions, the electrostatic, Van der Waals and H-bond interactions being almost simultaneously optimized. The full mechanism of formation of the newly grafted PAAm-g-CHT is summarized in Scheme 4. The spectral analysis also corroborated with the mechanistic simulation in terms of functional groups involved in coupling and complexation (FTIR structural variation analysis) and in terms of the degree of thermal stability within the copolymer structure which strengthens the experimental and computational correlation.

## 3. Experimental Section

### 3.1. Materials

Polyacrylamide (PAAm) (Mw = 5 × 10^6^ − 6 × 10^6^ g/mol), the synthetic non-ionic polymer, was supplied by Fluka Biochemika (St. Louis, MO, USA). Acrylamide monomer (AAm), potassium persulphate (KPS) (>98% purity) and chitosan (CHT) low Molecular mass (50–150 kDa) were purchased from Sigma-Aldrich (Sigma-Aldrich, St. Louis, MO, USA). PC12 neuronal cell lines were used as a model system for primary neuronal differentiation, derived from *Rattus norvegicus pheochromocytoma* and were purchased from the Health Science Research Resources Bank (HSRRB, Osaka, Japan). All other reagents used were of analytical grade and were employed as received.

### 3.2. Synthesis of Polyacrylamidated Chitosan Using Monomer (AAm-g-CHT)

Chitosan (CHT), polysaccharide backbone, was dissolved in 25 mL of a 1% *v*/*v* acetic acid aqueous solution via agitation overnight. After complete solubilization of CHT, the solution was decanted into a 150 mL reactor equipped with a N_2_ inlet and stirred for 30 min. Thereafter, the desired quantity of acrylamide monomer (AAm) and potassium persulphate (KPS) initiator were added to the solution maintained at 50 °C. The mass ratio of CHT:AAm was 1:4 and KPS:AAm was 1:5. After 6 h of reaction, polymerization was stopped by the addition of hydroquinone and the AAm-g-CHT was precipitated in an excess of acetone. The product obtained was further purified by Soxhlet extraction using 70% methanol as a solvent and finally dried at 40 °C in a vacuum oven (Vacuum Drying Oven “VACUTERM” EV-50, Raypa, Barcelona, Spain) for 48 h. The final grafted polymer was pulverized and fabricated into scaffold. For preparing the scaffold, a solution of AAm-g-CHT, equivalent to a 2% *w*/*w* CHT, was prepared in 0.2 M acetic acid. The solution was then decanted into 3 mL Teflon injection moulds (9 mm diameter) and frozen at −20 °C. The polymeric cylinders were removed after 6 h and immediately frozen at −80 °C overnight before being lyophilized (FreeZone^®^ 2.5, Labconco^®^, Kansas City, MS, USA) at 25 mtorr for 48 h. Thereafter, the cross sections of the scaffold were subsequently sputter-coated with gold for Scanning Electron Microscopy (SEM) analysis (Phenom™ Desktop SEM, FEI Company, Hillsboro, OR, USA). SEM images of the scaffold were quantitatively processed on Mathematica™ 8.0 (Wolfram Research, Champaign, IL, USA) using a sequential procedure of blurring, color-quantizing and generating an image histogram. Initially, the area of interest was restricted to image content of the scaffold.

### 3.3. Synthesis of Polyacrylamidated Chitosan Using Polymer (PAAm-g-CHT)

Chitosan was dissolved in deionized water (25 mL degassed) containing 1% *v*/*v* acetic acid in a reactor (with a N_2_ inlet) placed in a water bath preset at 50 °C. PAAm was dissolved in deionized water (5 mL degassed) in a separate flask. Upon reaching 50 °C, the PAAm solution and desired quantity of potassium persulphate (KPS) were added to the chitosan solution and the resulting mixture was allowed to react under a N_2_ nitrogen atmosphere. The graft copolymerization process was allowed to occur for 6 h at 50 °C and then terminated by adding hydroquinone and purified as above. The mass ratios of CHT:PAAm was 1:1 and KPS:PAAm was 1:1. The PAAm-g-CHT scaffold was synthesized and quantified using Mathematica™ 8.0 as discussed in previous section.

### 3.4. Cell-Culture and Cell-Seeding for *Ex Vivo* Tissue Engineering Evaluation

PC12 neuronal cells were grown in RPMI-1640 media (with l-glutamine and sodium bicarbonate) supplemented with 5% fetal bovine serum (heat inactivated), 10% horse serum (heat inactivated), 1% penicillin (100 IU/mL) and streptomycin (100 μg/mL) in an incubator with a humidified atmosphere (RS Biotech Galaxy, Irvine, UK) and 5% CO_2_ at 37 °C. Cells were grown in 75 cm^2^ cell-culture flasks in a monolayer to 80%–90% confluency and seeded into 96-well cell-culture plate at a seeding density of approximately 10,000 cells per well. Prior to cell-seeding, the AAm-g-CHT and PAAm-g-CHT scaffold were sterilized with 75% ethanol, washed twice with sterilized PBS buffer solution (pH 7.4) and finally rinsed in cell-culture media.

### 3.5. Determination of Grafting Parameters

Grafting parameters such as the Grafting efficiency (GE) and Grafting Ratio (GR) were calculated using Equations 5 and 6.

(5) $$\text{GE}=\left[\frac{\text{weight}\;\text{of graft copolymer}-\text{weight of initial chitosan}}{\text{weight of graft copolymer}}\right]\times 100$$

(6) $$\text{GR}=\left[\frac{\text{weight of graft copolymer}-\text{weight of initial chitosan}}{\text{weight of initial chitosan}}\right]\times 100$$

### 3.6. Determination of the Approximate Molecular Mass of the Grafted Polymer

The Mark-Houwink Equation (Equation 7) was used for the comparative profiling of the viscometric average molecular mass of the graft copolymers:

(7) $$[\eta ]={\text{KM}}^{\alpha }$$

Where, K and α are constants for a particular polymer/solvent/temperature system. For PAAm the values of K and α were 6.31 × 10^−5^ and 0.80, respectively [37]. Viscosity measurements of the 0.01% aqueous solutions of the graft copolymers were conducted on a Modular Advanced Rheometer system (ThermoHaake MARS Rheometer, Thermo Fischer Scientific, Karlsuhe, Germany).

### 3.7. Polymeric Structural Variation Analysis

Attenuated Total Reflectance-FTIR (ATR-FTIR) analysis was performed on the native polymers (PAAm and CHT) and the grafted copolymers (AAm-g-CHT and PAAm-g-CHT) to evaluate, ascertain and compare the structural transformations. ATR-FTIR spectra were recorded on a Perkin Elmer Spectrum 2000 FTIR spectrometer with a MIRTGS detector (PerkinElmer Spectrum 100, Llantrisant, Wales, UK), using an ATR-FTIR cell and a diamond crystal internal reflection element. Samples were analyzed at a wavenumber range of 650–4000 cm^−1^ with a resolution of 4 cm^−1^ and 64 scans per spectrum.

### 3.8. Exothermic and Endothermic Mapping of the Grafted Polymers

Comparative DSC analyses were performed on AAm-g-CHT and PAAm-g-CHT using a Mettler Toledo, DSC1, STAR^e^ System (Schwerzenback, Switzerland) at a heating rate of 10 °C/min from −10 to 325 °C under a constant flow of N_2_ gas. Accurately weighed samples (10–15 ± 0.1 mg) were placed into a covered aluminum sample holder with a central pin hole. Indium metal (99.99%) was used to calibrate the DSC modulus in relation to temperature and enthalpy. An empty sample holder was used as reference and experimental runs were performed by heating the samples from −10 °C up to 125 °C with a constant isotherm for 15 min. New samples were re-weighed and heated from −10 °C up to 325 °C. DSC thermograms were subsequently compared for transitions in thermal events.

### 3.9. Establishment of the Reactional Profile and Mechanisms via SLAS

All modeling procedures and computations, including energy minimizations in Molecular Mechanics, were performed using HyperChem™ 8.0.8 Molecular Modeling Software (Hypercube Inc., Gainesville, FL, USA) and ChemBio3D Ultra 11.0 (CambridgeSoft Corporation, Cambridge, UK). The decamer of acrylamide (PAAm) was archetyped using ChemBio3D Ultra in its syndiotactic stereochemistry as a 3D model, whereas the structures of chitosan (10 glucosamine saccharide units-CHT) was built from standard bond lengths and angles using the Sugar Builder Module on HyperChem 8.0.8. The structure of K_2_S_2_O_8_ (KPS) was constructed with natural bond angles. The models were primarily energy-minimized using the MM+ Force Field algorithm and the resulting structures were once again energy-minimized using the AMBER 3 (Assisted Model Building and Energy Refinements) Force Field algorithm. The conformer having the lowest energy was used to develop the polymer-polymer and polymer-KPS complexes. A complex of one polymer molecule with another was assembled by parallel disposition and the energy-minimization was repeated to generate the final models: CHT-KPS_4_, PAAm-KPS_4_, CHT-PAAm_2_ and CHT-PAAm_2_-KPS_4_ (digits in the subscript represent the number of molecules of a moiety modeled). Full geometrical optimization was conducted in vacuum employing the Polak–Ribiere Conjugate Gradient method until an RMS gradient of 0.001 kcal/mol was reached [38].

## 4. Conclusions

Anisotropic porous scaffold architectures for neural tissue engineering were obtained using a new grafting approach to fabricate PAAm-g-CHT for the first time. The semi-synthetic copolymer was successfully synthesized via a persulphate-initiated degradation free radical mechanism using PAAm instead of AAm monomers. The PAAm-g-CHT formed had higher molecular mass with superior grafting efficiency and neurodurability. The ATR-FTIR and DSC results confirmed the synthesis of PAAm-g-CHT and AAm-g-CHT and the structural and thermal transitions were similar between the copolymers with no significant variations proving optimal fabrication. Molecular Mechanistic simulations further aided in deducing the schematic mechanisms of the new graft copolymerization reaction. The high molecular mass polyacrylamidated chitosan exhibited promising neurocompatibility due to enhanced robustness, hydrophilicity and high surface charge with far-reaching use in specialized neural tissue engineering applications such as neural implants and conduits.

## Figures and Tables

**Table 1:** Intrinsic viscosity and molecular weight details of graft copolymers.

Graft Copolymer	GE a (%)	GR b (%)	IV c [η] [dL/g]	M_v_^d^ (×10^6^)
CHT-g-AAm	83	178	3.901	1.22
CHT-g-PAAm	92	263	5.231	1.63

a Grafting efficiency;

b Grafting ratio;

c Intrinsic viscosity;

d Viscometric average molecular weight.

**Table 2:** Energy attributes calculated for the optimized geometrical preferences of *in silico* complexes comprising chitosan, polyacrylamide and potassium persulfate.

Compound	Energy (kcal/mol)					
	
	Steric energy a	ΔE_binding_^b^	LDF c	Δldf d	H bond e	Ionic f
CHT g	35.556	-	13.323	-	0	−24.697
PAAm h	10.357	-	−5.072	-	−0.035	0
KPS_4_^i^	238.832	-	−5.776	-	0	0
CHT-KPS_4_^j^	239.153	−35.235	−27.503	−35.05	−0.0879	−32.394
PAAm-KPS_4_^k^	207.784	−51.762	−39.943	−29.095	−3.039	0
CHT-PAAm_2_-KPS_4_^l^	202.903	−92.199	−88.755	−86.158	−5.927	−33.481

a Minimized global energy for an optimized structure;

b ΔE_binding_ = E(Host.Guest) − E(Host) − E(Guest);

c London dispersion forces due to non-bonded/van der waals interatomic distances;

d Δldf = E(Host.Guest)_ldf_ − E(Host)_ldf_ − E(Guest)_ldf_;

e Hydrogen-bond energy function;

f Ionic energy arising from electrostatic interactions;

g Chitosan;

h polyacrylamide;

i Potassium Persulfate (four molecules);

j Chitosan complexed with four molecules of Potassium Persulfate;

k Polyacrylamide complexed with four molecules of Potassium Persulfate;

l Chitosan complexed with two molecules of polyacrylamide and four molecules of Potassium Persulfate.
